# Responding to fluctuations in public and community trust and health seeking behaviour during the COVID-19 pandemic: a qualitative study of national decision-makers’ perspectives in Guinea and Sierra Leone

**DOI:** 10.1186/s12889-024-20181-w

**Published:** 2024-10-04

**Authors:** Habibata Baldé, Brogan Geurts, Hanna-Tina Fischer, Sara Menelik-Obbarius, Ibrahima Kaba, Vitali Merhi, Karoline Stein, Viorela Diaconu, Thurid Bahr, Heide Weishaar, Alexandre Delamou, Abdul Karim Mbawah, Charbel El-Bcheraoui

**Affiliations:** 1 Centre d’Excellence d’Afrique pour La Prévention et le Contrôle des Maladies Transmissibles (CEA-PCMT), Université Gamal Abdel Nasser, Conakry, Guinea; 2 Evidence-Based Public Health Unit, Center for International Health Protection, Robert Koch Institute, Berlin, Germany; 3 College of Medicine and Allied Health Sciences (COMAHS), University of Sierra Leone, Freetown, Sierra Leone

**Keywords:** Trust, Essential health services, Health systems resilience, Communication, Community engagement, Pandemic preparedness, Qualitative

## Abstract

**Background:**

The level of trust in health systems is often in flux during public health emergencies and presents challenges in providing adequate health services and preventing the spread of disease. Experiences during previous epidemics has shown that lack of trust can impact the continuity of essential health services and response efforts. Guinea and Sierra Leone were greatly challenged by a lack of trust in the system during the Ebola epidemic. We thus sought to investigate what was perceived to influence public and community trust in the health system during the COVID-19 pandemic, and what strategies were employed by national level stakeholders in order to maintain or restore trust in the health system in Guinea and Sierra Leone.

**Methods:**

This qualitative study was conducted through a document review and key informant interviews with actors involved in COVID-19 and/or in malaria control efforts in Guinea and Sierra Leone. Key informants were selected based on their role and level of engagement in the national level response. Thirty Six semi-structured interviews (16 in Guinea, 20 in Sierra Leone) were recorded, transcribed, and analyzed using an inductive and deductive framework approach to thematic analysis.

**Results:**

Key informants described three overarching themes related to changes in trust and health seeking behavior due to COVID-19: (1) reignited fear and uncertainty among the population, (2) adaptations to sensitization and community engagement efforts, and (3) building on the legacy of Ebola as a continuous process. Communication, community engagement, and on-going support to health workers were reiterated as crucial factors for maintaining trust in the health system.

**Conclusion:**

Lessons from the Ebola epidemic enabled response actors to consider maintaining and rebuilding trust as a core aim of the pandemic response which helped to ensure continuity of care and mitigate secondary impacts of the pandemic. Monitoring and maintaining trust in health systems is a key consideration for health systems resilience during public health emergencies.

## Background

Access to essential health services is a key element in protecting population health and the effective functioning of any health system. The availability of healthcare services and their subsequent delivery and uptake is relevant to the system’s effectiveness but dependent on a number of interrelated factors, including the acceptance by providers and populations and their trust in the system [1]. Trust is a critical element of social relations, affecting the decision-making process and the behavior adopted in response to different situations [2]. The concept has been broadly studied and conceptualized by researchers in various forms, particularly in health systems [3–6]. The central common element in the multiple definitions is the conception that trust is the “acceptance of a vulnerable situation in which the trustee believes that the trustor will act in the trustee’s best interest” [3, 6–8]. Trust as such has been further understood as the ‘‘relational notion or psychological state that influences individuals’ willingness to act on the basis of the words, motives, intentions, actions and decisions of others under conditions of uncertainty, risk or vulnerability’’ [5]. Trust reaches from the individual level to the wider community and public population levels in which people identify, belong, or are grouped and further extends to health system, institutional, and government levels.

Public managers and institutions have a significant role in building trust with the public and specifically play a role in mitigating perceived uncertainty or vulnerability [4]. Publics benefit from high perceptions of trust in powerful institutions which they may depend on, as it may lead to feeling a sense of security, safety, and possible meaning or common identity [9]. When public trust is considered high, institutions or individuals in power may also benefit from lower levels of scrutiny and higher levels of compliance. Therefore, institutional trust formation may have potential for conscious or unconscious ulterior motives, yet still requires decision makers and individuals in power to understand and recognize the importance and function of trust within society. If levels of trust remain too high or too low negative consequences may ensue for either side [4, 9]. Mutually beneficial trust between institutions and populations therefore particularly requires engagement with and participation from the public with institutions or decision makers [9].

Public levels of trust in health systems are often in a dynamic state of flux during public health emergencies. Trust informs and guides communities’ health-seeking behavior [10] and adoption of prevention measures [11–13]. Changes in trust pose challenges for providing adequate health care and preventing the spread of disease. Trust has been a critical component in managing health emergencies [10, 12, 14] and has been proposed as an eighth core component of the WHO health systems responsiveness framework [15]. “Effective utilization of, and trust in, healthcare services is [additionally] essential to facilitate early case detection” [14]. Lack of trust has been identified as a reason for the low-uptake of preventive and containment measures [11, 13] and under-utilization of health services in emergency and post-emergency settings [10, 16]. Pre-existing mistrust in governments and health systems, which was exacerbated during the 2013–2016 Ebola outbreak in West Africa, has been repeatedly cited as a key factor that contributed to the initial failure to contain the outbreak. [12, 14, 16, 17]. This failure was particularly represented by fear and misconceptions related to a lack of trust in the health system [10, 16, 18] and highlighted the need for decision makers to be mindful of trust in order to ensure populations continue to seek health services during such crises. This has included the increasing recognition of the vital role community engagement and social participation play within health systems and particularly during public health crises [19, 20]. This recognition further extends to the role health care workers play, particularly as health care workers, including community health workers (CHWs), are both members of the public and communities in which they live and work as well as part of the health system.

Guinea and Sierra Leone were severely affected by the Ebola epidemic in 2013–2018 with 3,811 cases and 2,543 deaths in Guinea and 14,124 cases and 3,956 deaths in Sierra Leone respectively [21]. Both countries faced a number of challenges during and following the epidemic due to a lack of perceived trust in the health system and government, including a marked reduction in the utilization of health services [10, 16, 22, 23]. Control efforts were hindered by community resistance, where cases of vandalism of health facilities and violence against health workers were documented in Guinea [24]. Interventions such as the use of national hotlines, Ebola treatment centers, and chlorine sprays by health workers in Sierra Leone initially faced resistance. There was widespread skepticism and mistrust regarding the effectiveness of these interventions, mirroring doubts about the existence of Ebola itself. Ebola treatment centers were viewed as places of inevitable death, and there were misconceptions such as the belief that calling the emergency hotline would lead to the demise of the person in need or that the chlorine sprayed by ambulance workers was harmful to the sick [18, 25]. Several reasons have been associated with community resistance during the Ebola epidemic, including misconceptions about the disease and failures in the emergency response that undermined the public’s confidence in the response. Inadequate and ineffective communication about Ebola and its public health and social measures (PHSM), the failure to implement response interventions that were tailored to local customs and social norms, and the lack of or limited involvement of communities in response activities limited public trust and hindered efforts to control the epidemic [16, 23, 24, 26].

Following the painful experiences of the Ebola epidemic, communities remained distrustful towards healthcare providers [27, 28]. Post-Ebola epidemic health facility attendance decreased compared to the pre-epidemic period, with one of the causes being a lack of trust in healthcare staff and the healthcare system as a whole [10, 29, 30]. Several post-Ebola epidemic interventions centered their efforts on rebuilding trust between communities, the state, and the health system. Key interventions focused on rebuilding trust and reducing stigma by engaging local leaders and other community-based volunteers in communication and outreach activities [31]. CHWs were particularly shown to have played an essential role in restoring trust through the implementation of activities such as door-to-door or group sensitization, resumption of primary health care provision, and referral of patients to health facilities [28]. While CHWs were shown to have been key in restoring trust post-Ebola due to their position within their communities, they were also noted as a source of uncertainty at the outset of the epidemic due to their additional connection to the health system [23]. This meant trust in CHWs similarly fluctuated throughout the epidemic and in the post-epidemic period. In both countries, health seeking behaviors and trust in the health system has however been noted to have improved post-Ebola. While evidence is limited, in Guinea, it is not certain if levels of trust have fully reached or exceeded the levels of trust from the period prior to the Ebola epidemic [30], while in Sierra Leone levels of trust and health facility attendance may have exceeded pre-Ebola levels [32, 33].

During the COVID-19 pandemic, public trust in health systems and the ability to respond to the needs of the population was tested once again in Guinea and Sierra Leone [34]. The global emergence of COVID-19 brought a new set of PHSMs which, along with ongoing difficulties in accessing and providing essential services, put the public’s trust in the health system and government to the test [35–38]. Evidence on the maintenance of trust during the COVID-19 pandemic, especially in low- and middle-income countries (LMIC), is limited [13, 39]. Existing studies have largely focused on the public’s compliance with PHSMs and role of trust in governments [11, 12, 40, 41], and the interpersonal relationships between patients and providers [10, 17]. Less attention has been given to public and community trust in the health system [39] and what were the corresponding actions taken by decision makers.

In April 2020, the African Centre for Disease Control and Prevention (ACDC) provided guidance for African Union Member States on the continuation of essential health services during the COVID-19 pandemic citing the importance of learning from the preventable deaths due to measles, malaria, HIV/AIDS, and TB during the West African Ebola epidemic. Evidence on how essential health services were maintained during the COVID-19 pandemic is currently lacking, either from the community or decision makers perspective [42]. Previous experiences with Ebola demonstrated the importance of trust for the effective provision of essential health services during health crises. Investigating the role of trust in the health system during COVID-19 in Guinea and Sierra Leone can help to gain a further understanding of the relationship between trust and the utilization of essential health services during public health emergencies.

In this study we sought to investigate national level decision makers perceptions of public trust and what was thought to have influenced trust in the health system and use of essential services during COVID-19. As previous outbreaks have demonstrated an unexpected and substantial decrease in public trust can have a negative impact on the response. Therefore, we sought to investigate how the perceptions of national policy makers regarding changes in trust and health-seeking behavior influenced the strategies, measures or decisions that were taken to mitigate potentially adverse consequences during COVID-19. We sought to understand this relationship within the context of public health emergencies when explorations of trust at the community or population level may be limited yet rapid decisions and assessments may be necessary. We chose malaria services as a case study to explore health-seeking behavior and essential services, as perceived by national malaria and COVID-19 decision-makers. The reason behind our choice is due to malaria being a priority endemic disease that cuts across community prevention to tertiary treatment levels of the health system. This makes malaria a key priority for national decision makers during health emergencies. The ACDC’s Africa Health Strategy 2016–2030 further specifically considers malaria as a top priority endemic disease whose services should continue uninterrupted during a pandemic [43].

## Methods

We used qualitative research methods to explore the perceived impact of the COVID-19 pandemic on public trust in the health system and the corresponding mitigation measures enacted in post-Ebola Guinea and Sierra Leone, from the perceptions of national level decision makers. Malaria interventions and services were used as a case study to explore one cross-cutting area of the health system. We understand trust in this study as a relational concept based on the trustee’s specific expectations of the trusted person, such as a patient’s or the public expectation that healthcare staff or health system will act in one’s best interest. We further understand health care workers, including community health workers, as individuals who work in the health system but are also members of the general public and the communities in which they live. We conducted a document review and collected data through key informant interviews. As part of a larger study key informant interviews were coupled with a social network survey and were followed by a subsequent quantitative phase, including a health facility and household survey and secondary data analysis. Results from the larger study are presented elsewhere [44].

### Document review

We reviewed key documents for information on the epidemiological situation of malaria and COVID-19 in Guinea and Sierra Leone before and during the COVID-19 pandemic period We searched the websites of key malaria and COVID-19 stakeholders in Guinea and Sierra Leone including the relevant Ministries of Health, World Health Organization (WHO), USAID President’s Malaria Initiative (PMI) and the Global Fund to Fight AIDS, Tuberculosis and Malaria as well as obtained specific documents through local academic and governmental networks such as reports or policy documents that were not publicly available. The document review provided an overview of malaria programs and the COVID-19 timeline, including the scope of malaria activities and PHSM implemented during COVID-19. The review additionally provided an initial identification of relevant stakeholders within each country and informed the development of the interview topic guide.

### Key informant interviews

In addition to the document review, we conducted scoping interviews and site visits in Conakry, Guinea and Freetown, Sierra Leone in October 2021 (IK, BG, HW, CEB in Guinea and AKM, HTF, FPM, CEB, in Sierra Leone). These scoping interviews and site visits along with the document review served to identify key stakeholders working on malaria elimination or COVID-19 prevention and control in Guinea and Sierra Leone as well as to inform the development and contextualization of interview topic guides as well as surveys undertaken as part of the larger study. Site visits included national malaria control programs, regional/district health administration offices, and national and regional hospitals and health centers. Scoping questions focused on the malaria prevention and care cascade within the context of COVID-19 as well as who are the key individuals involved at the administrative and policy levels.

We conducted semi-structured interviews in March 2022 with national level stakeholders and decision makers leading the response to COVID-19 and/or engaged in malaria elimination efforts in-country in Guinea and Sierra Leone. These included representatives from national and international non-governmental organizations (NGO), government representatives, international organizations working in-country, and bilateral and multi-lateral funders supporting COVID-19 response and malaria elimination efforts. Key informants were sampled using a purposeful and snowball approach based on their role and level of engagement in the national level response to COVID-19 and malaria elimination. Purposeful sampling was based on availability and organization rather than specific individuals, while snowball sampling was elicited at the end of each interview to ensure key organizations were not overlooked during the initial sampling process. Topic guides and interviews focused on four areas of interest: (1) the overall response to COVID-19, (2) the impact of COVID-19 on malaria elimination activities and corresponding health system elements, (3) population health-seeking behavior during COVID-19, and (4) perceptions of public trust in the health system. Topic guides were slightly adjusted during data collection based on emerging data and the type of key informant.

Potential key informants were contacted by the local principal investigator or research lead (AD or IK in Guinea and AKM in Sierra Leone) via email and phone. Key informants were provided with an overview of the study objectives and were informed about the voluntary nature of the study as well as given the opportunity to ask questions prior to arranging an interview. All individuals or organizations invited to take part in an interview accepted with the exception of two who could not participate due to scheduling difficulties (one in Guinea and one in Sierra Leone respectively). Key informants were briefed about the study and provided their written and verbal informed consent. Organization level data was collected for the descriptive purpose of the sample (Table 1). Interviews in Sierra Leone were conducted in English while interviews in Guinea were conducted in French, with the exception of one interview conducted in English. Interviews were conducted by local researchers (AD, IK, HB in Guinea and AKM, LSB in Sierra Leone) and German-based researchers (CEB, TB in Guinea and HTF, BG in Sierra Leone). With the exception of one interview in Sierra Leone, at least one interviewer and one note taker were present in each interview. All interviews took place in person with the exception of one interview in Guinea which took place online due to scheduling feasibility. All interviews were audio recorded and lasted on average 43 min. We applied the Standards for Reporting Qualitative Research (SRQR) guidelines [45].

**Table 1:** Summary of key informants

Type of Key Informant	Guinea (*n* = 16)	Sierra Leone (*n* = 20)	Total (*n* = 36)
Non-governmental Organization (NGO)	5 (31%)	8 (40%)	13 (36%)
National Government	8 (50%)	8 (40%)	16 (44%)
International Organization	-	3 (15%)	3 (8%)
Bilateral / multi-national Funder	3 (19%)	1 (5%)	4 (11%)

### Data analysis

All interviews were transcribed by members of the research team in the language they were conducted. Interviews conducted in French were translated to English by two members of the research team and cross-checked between the two translators. Identifying information, such as names, were removed prior to analysis.

Using an inductive and deductive framework approach to thematic analysis interview, 34 transcripts were analyzed [46, 47]. The framework approach is particularly suited to applied policy research in the health sector [48]. Following transcription, an initial codebook was developed based on the topic guides and WHO’s health system building blocks [49]. In parallel, the data analysis team familiarized themselves with the transcripts. The codebook was then refined using in vivo codes identified and defined by the informant’s wording [46]. Development of the codebook was an iterative process involving piloting the codebook on select transcripts to refine the codes until consensus was reached among the research team. The final codebook included a definition for each code, specifications for when to use it, and specifications for when not to use it. All transcripts were independently coded using NVivo (1.7) qualitative data management software, by at least two coders. Subjectivity memos and tests of inter-rater reliability were conducted to ensure consistency across the coding team. Given the diversity of the analysis team across cultural contexts, this was deemed as an appropriate step to ensure consistency [50].

The process of identifying categories and themes was iterative and involved refining categories and themes in a collaborative process. Saldana’s codes to theory model was used through constructing summaries [51]. Triangulation and agreement across actors were a central focus of the analysis and identification of themes. An Excel file was used throughout the process to track changes that were made to the labels of the codes, categories, and emerging themes as well as to develop code trees and cross-linkages across categories and themes. A corresponding data display was developed to visualize and map cross-cutting categories and themes.

Prior to the publication of results, a stakeholder workshop was held in each country to discuss, validate and disseminate the overall study results among study participants, health authorities and relevant policy makers, academics and international partners. Preliminary results were well-received and validated by study participants and were in-line with our analysis and recommendations. In two cases participants provided additional details that were not captured in the data. In Guinea participants highlighted strategic and resource components related to the country-wide distribution of insecticide treated malaria nets during COVID-19, while in Sierra Leone participants detailed taking a similar approach as during the Ebola epidemic to provide mass treatment for malaria during emergencies.

## Results

We interviewed a total of 36 national level stakeholders involved in the response to COVID-19 and implementation of malaria control programs (Table 1).

We identified three overarching themes which were described by key informants as influencing changes in trust and health seeking behavior due to COVID-19 and the corresponding mitigation measures: (1) reignited fear and uncertainty among the population, (2) adaptations to sensitization and community engagement efforts, (3) building on the legacy of Ebola as a continuous process. Nearly all respondents described an initial drop in health facility utilization, irrespective of malaria services or otherwise, at the onset of the pandemic due to fear of infection, fear of diagnosis and isolation, and general mistrust. This fear and mistrust were said to have emerged among both the population and healthcare workers and was informed by experiences during the 2013–2016 Ebola outbreak. Key informants described actions were promptly taken to mitigate against fears and mistrust. Respondents described three main actions that were perceived as important for addressing fear and mistrust during the first two years of COVID-19: communication, community engagement, and on-going support to health workers. Rationale for these mitigation measures was further described as influenced by lessons learned during the 2013–2016 Ebola outbreak. This led to a recognition for a need for a continuity in trust sustaining measures. Table 2 provides an overview of the themes and sub-themes.

**Table 2:** Overview of themes and categories

Overarching Themes	Sub-themes
Reignited ear and uncertainty among the population	*The memory of Ebola*
	*A fear of COVID-diagnosis or misdiagnosis*
	*A lack of PPE contributed to fear of infection*
Adaptations to sensitization and community engagement efforts	*Communication and sensitization were intensified*
	*Important role of community health workers in the response*
Building on the legacy of Ebola as a continuous process	

### Reignited fears and uncertainty among the population

Respondents described what they considered an initial decrease in health facility utilization and attendance during the onset of the pandemic and at times during subsequent waves of infection, irrespective of malaria specific services. Respondents attributed this change to memories of the 2013–2016 Ebola epidemic which invoked fears and uncertainty on behalf of facility healthcare workers and patients alike. This fear and uncertainty intensified as misinformation and rumors circulated, particularly in regards to isolation and the severity of the disease. Health care workers attendance was further described as influenced by insufficient availability of personal protective equipment (PPE) and a fear of infection. In Sierra Leone, where lockdowns were put in place as a PHSM to slow the spread of disease, respondents described this had an effect on facility attendance due to movement restrictions and restricted economic activities. According to one national NGO respondent this contributed to a general “*type of pandemonium*” *(NGO Sierra Leone, KII014)* and panic among the population for accessing limited daily necessities and resources and an additional perceived unavailability of (safe) health services were they to fall ill.

### The memory of Ebola

The memory of Ebola framed the expectations and rationale surrounding visiting a health facility during COVID-19. Respondents described that the populations in both countries associated going to the facility with a risk of being infected with COVID-19 and being automatically isolated. Due to the initial unknown severity of COVID-19, some respondents associated this association with a further concern that those infected may not return from the facilities, as was experienced during the Ebola epidemic. The fear of being diagnosed extended beyond the severity of the disease. Respondents described that mandatory isolation was seen as threatening one’s livelihood given that people had few economic and survival options if they were isolated. An issue that was also experienced during the economic impact of the Ebola epidemic. One international organization representative reflected,

“*What I can see from afar is that at the beginning, people were afraid to go and be diagnosed with [COVID-19] because they thought that it could be like Ebola. Then maybe in the beginning there was a lot of self-medication so they wouldn*’*t be in isolation.*”* (International Organization, Guinea, KII015)*

### A Fear of COVID-19 diagnosis or misdiagnosis

Respondents further described that the population expressed mistrust in the health system, in which they were reported to fear that someone would be automatically diagnosed or wrongfully diagnosed with COVID-19 simply by visiting a health facility. This was in part influenced by misinformation and rumors, but substantiated by symptoms of fever and headache which are common symptoms to seek services for, particularly for malaria. Communities were described by some respondents to be confused about the disease since little information was available at the beginning of the pandemic. The spread of rumors, unsubstantiated information and the lack of information on specific aspects of the disease, such as its asymptomatic nature, were said to have contributed significantly to the uncertainty and fear among the population. A government respondent in Sierra Leone and Guinea both exemplified,

“*The Ministry and [governmental COVID response agency] itself had to go out to convince people to visit the hospital, because people were always apprehensive that if they come to the hospital they will tell them that they have COVID and they will keep them in the hospitals, so a lot of people are not actually visiting the health centers.*”* (Government, Sierra Leone, KII012)*

“*In any case [COVID-19] changed the behavior of the population in terms of consulting public services for fear of being declared positive for Covid-19. Some people even refrained from consulting, they preferred to stay at home and not consult, not to go to a health structure.*”* (Government, Guinea, KII002)*

### A lack of PPE contributed to fear of infection

The lack of sufficient PPE and training on COVID-19 and infection prevention and control measures was described as contributing to health care workers’ fears. Working without the skills and resources that were needed was perceived by respondents as a major demotivating factor for staff continuing to provide care since they felt unprotected and at high-risk. Key informants had described this as indicative as some health care workers were described as refusing to return to work without being equipped with proper PPE. In Sierra Leone some respondents further described a number of publicized COVID-19 deaths of health workers that were seen as further contributing to the fear of infection among health care workers particularly. Ensuring health care workers had adequate PPE was however described by NGO’s as a challenge due to the dependence of the national supply chains on the global supply chain. A pre-existing common challenge to move commodities to the ‘last mile’ was described as being exacerbated by local and global PHSMs that restricted movement and closed borders. A respondent from an international NGO in Guinea described,

“*The big challenge was that, how to make sure that the staff and those in need could have the material they needed, that was the big challenge, and it took a lot of time… The pandemic started, we only waited six months, nine months for the first equipment, the materials for people to be able to protect themselves and work.*”* (NGO, Guinea, KII013)*

Key informants further perceived health care workers fears and lack of motivations to have influenced trust in the health system and health seeking behavior of the communities in which health care workers live. Respondents understood that if health care workers themselves did not feel safe to attend work, why would the population. Another respondent from the Guinean government further elucidated,

“*When we started putting [PPE] in place, confidence started to return [among health care workers …] then [confidence in] the health facility [and then among] the communities also since the healthcare providers were now available […] it started changing, getting back to normalcy but it took some time.*”*(Government, Guinea, KII005)*

### Adaptations to sensitization and community engagement efforts

Respondents described specific mitigation measures that were put in place in order to address the fears and mistrust that had emerged. Almost all respondents reported that the drop-in health facility utilization was temporary in nature, and that health facility attendance rates had returned to pre-pandemic levels in both countries at the time of the interviews. Key informants understood community sensitization and support for healthcare workers as key mitigation measures for addressing fear and trust in the health system.

### Communication and community sensitization

Community members were reported to have started to return to seek healthcare services once the situation appeared to be not as serious or life threatening as perceived in the beginning of the pandemic. Rebuilding trust and ensuring the return to health facilities, according to respondents, was strongly influenced by the initiation and intensification of health communication and community sensitization activities, including the adaptation of existing health campaigns to include COVID-19 messages, such as preventative malaria campaigns (e.g. mass distribution of long lasting insecticide treated nets, seasonal malaria chemoprevention for high risk groups). This was described as key to fighting rumors in a time of uncertainty and a surge in information globally. People began to feel reassured and less afraid as they realized COVID-19 was less severe than Ebola and trusted services returned. At the same time, it was noted that the sensitization of health care workers was a first priority for decision makers.

### Important role of community health workers in the pandemic response

Respondents in Guinea highlighted that the policy for CHWs to come from the communities in which they serve played a crucial role in rebuilding trust and encouraging people to seek healthcare information and services during the pandemic. CHWs were quickly engaged in the COVID-19 response and were described as in scaling up sensitization activities and providing services within the community. In Guinea this included adapting malaria specific interventions to include COVID-19 sensitization, such as reducing close interactions, interaction time and sharing of utensils during the administration of malaria chemoprevention for children under 5. This was described as key to maintaining preventive services through specific individuals the community trusted while at the same time there was reluctance to go to a health facility. It was noted that the community members’ relationship with CHWs was not a new aspect of the COVID-19 response, but was an existing component of the health system and lasting impact from Ebola.

In Sierra Leone, informants described a ‘No touch policy’ that was mandated as a PHSM for CHW at the start of the pandemic. This restricted the ability for CHWs to continue to provide services beyond some forms of sensitization and referral to health facilities. Respondents iterated this put a complete halt to malaria services previously undertaken by CHW, such as the administration of rapid diagnostic testing and distribution of seasonal chemoprophylaxis. The was partly attributed to a challenge to train the sheer number of CHW on COVID-19 and equip them with adequate the limited PPE that was available. Concurrent to the COVID-19 pandemic, respondents described an on-going restructuring of the national CHW policy (Table 3). This resulted in the additional formal suspension of CHW activities.

**Table 3:** Reform of the policy on community health workers in Sierra Leone, as described by key informants

The overall restructuring of the community health worker policy was described as a long on-going process that had begun prior to COVID-19 and in part response to the Ebola epidemic. The reform of the policy was contributed to at least three main factors by respondents: 1) the approximate 16,000 CHWs were reported as too many for the Ministry of Health to manage 2) the functions of community health workers were not integrated across primary health care but soiled by funders and programs 3) requirements to be a CHW were deemed inconsistent and not suitable for an integrated approach, this primary included education level and gender balance. The reform in the new policy resulted in a temporary suspension of CHW and re-recruitment which included reabsorbing some CHW who met the new criteria. Payment of CHW during this process was described as paused and included needing to return donor funds due to not providing incentives for services distributed. Some CHWs, however, were described to have continued working due to their connections with healthcare workers and a hope that they would be paid or reabsorbed into the new system.	

As one international organization described, “*CHWs were not operating the way they should have*”*. (International organization, Sierra Leone KII09.2).* Additional key informants confirmed the situation would have been different if CHW had been able to continue to provide routine testing and treatment services for malaria as well as mobilize and engage with communities and ease uncertainty and fears surrounding COVID-19 or visiting a health facility. This was described as having a two-fold impact: similar to during the Ebola outbreak, the demand and the necessity to mobilize CHWs became clear and the CHW policy was fast-tracked. Ultimately resulting in a long awaited restructuring of the overall CHW policy which was seen as positive by respondents but unfortunately came at an inopportune time that was described as affecting trust during the initial response to COVID-19.

### Building on the legacy of Ebola as a continuous process

Respondents described how lessons related to responding to trust in health systems learned during the Ebola response in 2013–2016, had been reinforced during the COVID-19 response and informed their decision making. The importance of close relationships between health care workers and the communities in which they live and are part of, for example, was stressed. This reemphasized the need for on-going capacity and material support for health care workers and the importance to work with CHW that come from the local communities in which they serve and trust. Health care workers, including CHWs, and other community actors such as local leaders, were perceived as the driving force for successful communication and sensitization which led to the rebuilding of trust and health seeking behavior. The further quick engagement and involvement of CHW into the COVID-19 response in Guinea was seen as a driving force for ensuring trust in the system, while the limitation of CHW activities and roles in Sierra Leone was seen as a misjudgment by some. In Sierra Leone, the COVID-19 pandemic was described as providing the needed political push to fast-track, what was overwhelmingly described by respondents as a long-awaited and essential, restructuring of the CHW policy (Table 3). Respondents further emphasized the need to continue to strengthen the health system by maintaining consistent communication and building capacities of health care workers. When reflecting on the response, respondents emphasized that they learned the same lessons during COVID-19 as they did during the Ebola response and this reinforced their confidence in the decisions that were taken and had potential for future system-wide planning. As one government representative in Guinea reflected,

**“***One of the lessons is that the health system must continue to prepare for epidemics. When Ebola came, there was a time when there were a lot of things that were borrowed as good practices. But when Ebola went away, some of these good practices were no longer applied.*”* (Government, Guinea, KII011)*

Ultimately, respondents reflected on the importance of maintaining programs and resources which are deemed as trust building, such as well-supported community health workers programs, communication and infection prevention and control resource capacities. From Ebola and COVID-19 maintaining trust was seen as a continuous process which should take place outside of emergencies as well.

## Discussion

In this study we sought to understand the perceived impact of the COVID-19 pandemic on trust in the health system in Guinea and Sierra Leone through the lens of national COVID-19 and Malaria decision makers. Malaria was used as a case study to frame the perspective around a set of essential health services which cut across multiple levels of the health system. We identified key areas which were considered as important components of the corresponding mitigation measures to maintain and rebuild trust during a dynamic pandemic. Our results suggest that through instituting and reinforcing lessons learned during the 2013–2016 Ebola epidemic, it was deemed possible to quickly restore community and public trust and health seeking behavior to pre-pandemic levels after an initial dip at the start of the pandemic. Notedly previous experiences during the Ebola epidemic helped national level stakeholders and decision makers to recognize community and public trust as a key area for the successful response to COVID-19 and maintenance of essential health services [12, 14, 40, 52, 53]. During COVID-19, communication [54], community engagement [10, 18, 41], and the on-going support of health care workers [54] again became apparent as crucial components for maintaining trust in the health system.

### Maintaining trust and essential services

Our results support several lessons which can inform responsiveness to perceived changes in trust in health systems during a public health emergency or similar event with implications for health system performance. First and foremost, the importance of maintaining trust in health systems as well as ensuring the continuity of health service utilization during and after public health emergencies is key to managing outbreaks [10, 41]. Ensuring the continuity of health service utilization mitigates the secondary health impacts from an epidemic on on-going health system progress while also providing additional outbreak prevention and optimizing case detection [14]. Our findings regarding the decrease in service utilization due to fear and lack of trust in the health system are in line with several studies that have described similar phenomena in epidemic settings [10, 16, 52, 55], including Ebola, and during COVID-19 in Guinea and Sierra Leone [56, 57]. Studies have particularly identified parallels between the COVID-19 pandemic and the Ebola epidemic, including the widespread circulation of misinformation about the diseases, leading to fear of infection and distrust in healthcare systems and reduced utilization of healthcare services during these periods [58, 59]. Kreuk et al. reflect that one key lesson learned during the Ebola epidemic was that “health systems that earn the trust and support of the population and local political leaders by reliably providing high-quality services before crises have a powerful resilience advantage” [60]. There is a need to continuously monitor trust and health seeking behavior during and outside of health crises in order to anticipate potential changes in health seeking behavior and establish immediate mitigation plans [15, 52, 54]. Whereas our results reflect perceptions of national level decision makers, changes should be monitored at multiple levels, including routine facility attendance data and perspectives of communities themselves.

### Early risk communication

Second, maintaining trust includes the incorporation of risk communication early in the response, prior to the detection of a disruption in health seeking behavior or trust [61]. During the Ebola epidemic, the lack of information and the spread of rumors surrounding the disease contributed to public fear and the desertion of facilities by both providers and communities [16, 41] and the need for intensified risk communication became prominent [54]. It seems that lessons from Ebola contributed to a recognized need and an improvement in risk communication during COVID-19. Our results provide evidence of an immediate acknowledgement of the risks with regard to changes in trust at the beginning of the COVID-19 pandemic and a quick response by stakeholders in establishing a communication system to prevent a prolonged lack of trust in the health system, as occurred during the Ebola outbreak [23]. Our findings are in line with a study on the pandemic response to COVID-19 in Guinea which shows that stakeholders emphasized the rapid responsiveness, upscale, and integration of communication [61].

### Community engagement and community health workers

Third, community engagement and the mobilization and continuous involvement of CHWs are key in maintaining trust and promoting ownership of response measures [10, 52, 62, 63]. CHWs serve as a crucial bridge between communities and the healthcare system for maintaining trust and moving people towards behavior change, as they are integral members of both spheres [64–66]. The 72nd World Health Assembly adopted a resolution that recognizes that CHWs are further “indispensable to contribute to ongoing primary health care services during emergencies” and are a cost-effective mechanism [67, 68]. However, the prior existence of community health programs and community engagement activities does not ensure the effective involvement of communities in emergency health response activities [23, 69]. During the Ebola epidemic, CHWs were initially sidelined in community response interventions, with the recruitment of new Ebola-specific agents who were rejected by the populations with the messages they carried [23]. Including CHWs in the Ebola response was reported to be a late but successful strategy in restoring confidence, prompting the use of health services, and acceptance of established response measures [16, 23], as was similarly found during COVID-19 in our study. Rather, adequate and effective systems of community health programs have to be developed that firmly integrate CHWs as a full-fledged function into health systems and ensure basic salaries for these actors in accordance with the WHO’s 2018 *Health policy and system support to optimize community health worker programmers* [70, 71]. Establishing CHW programs is crucial as outreach services and facility-based services are likely to be the most disrupted in the early phases of a public health emergency, particularly in LMIC [55]. This remains of critical importance as CHW programs continue to gain traction yet remain fragmented and chronically underfunded across the African continent [72–74].

### Ongoing support of heath care workers

Lastly, the on-going support of health care workers, including community health workers, is crucial in order to maintain their trust in the system, including with and between communities. Lack of training and PPE can hamper health care workers’ willingness to provide and community members’ readiness to use health services. As seen during Ebola and COVID-19, it also reduces essential trust in the system’s ability to respond [16, 60, 63, 75]. Across the COVID-19 pandemic, health care workers have, globally as well as in Guinea and Sierra Leone, been described as feeling ill-prepared and ill-equipped to provide necessary services [76–78]. The fact that ongoing support for health care workers to “show up for work that might be difficult and dangerous […] begins with training …” was similarly acknowledged as a key learning during the Ebola epidemic [60]. WHO and other global health actors have thus highlighted the need for providing health care workers with the proper resources to respond to public health emergencies in a safer environment [34, 36, 76]. Disruptions in global medical supply chains of PPEs and medical equipment during COVID-19 have been well-documented across countries [55] and will require the establishment of resilient and contingency plans as service demand will inevitably be affected during a crisis [36–38, 55, 79]. Without this continued support health care workers and the public have room to lose trust in the health system. Building strategies to ensure the continued supply of resources and materials into emergency response plans is essential for supporting health care workers to provide services and duly contribute to trust in the system’s ability to provide essential services.

### Limitations

Our study has several limitations. First, we focused on policy and decision makers’ perceptions of changes in trust and health seeking behavior during COVID-19, and cannot draw direct conclusions about actual changes in trust the individual or community level without data collection at these two levels. However, our goal was to gain insight into how decision makers responded to rapidly evolving changes in perceived trust and facility utilization. Second, our sample included individuals in high and varying levels of power and is limited to their perspective within an emergency context. This perspective may have been influenced by socially desirable responses or may be somewhat detached from the actual experiences of trust as felt by the population. While we did not explicitly include a power lens to our analysis, we attempted to triangulate data between the different informants across sectors and outside of the national government to tease out potential bias or socially desirable answers. Third, we interviewed national level stakeholders and, therefore, cannot draw upon the nuances of changes and responses to trust that may be more specific at regional or local level. Forth, our sample of COVID-19 and malaria program actors does not necessarily represent all views within the health system. Additionally, interviews only captured a snapshot in March 2022, when the pandemic was mostly considered over in both countries. The use of qualitative methods, however, has helped to mitigate these limitations by exploring potential dynamic policy changes from multiple perspectives and providing the flexibility to iteratively adapt the research tool as the pandemic and situational context evolved. Lastly, selected key informants provided their recollections and perceptions of the most important factors influencing trust and decision making during the response. Although this perspective is limited by the absence of first-hand accounts from community members, it may still serve as valuable evidence for emergency situations like COVID-19, where rapid decision-making and leadership are required.

## Conclusion

The COVID-19 pandemic has reiterated that the trust of the public in health systems plays an ever more important role during public health emergencies. Likewise, it has shown the benefits of learning from previous experiences. In Guinea and Sierra Leone, lessons from the Ebola epidemic enabled response actors to consider maintaining and rebuilding trust as a core aim of the pandemic response which helped to ensure continuity of care and mitigate secondary impacts of the pandemic. Incorporating mechanisms to monitor and maintain public trust in the health system during public health emergencies remains an important consideration for maintaining essential health services and health systems preparedness and resilience in future public health emergencies. It would be worthwhile to research public trust from the perspective of communities to better understand reactive views regarding trust and related dynamics during and beyond periods of uncertainty, fear and crisis. Given that there is evidence that public trust in the system can ensure continuity in the use of routine services and compliance with PHSMs, it is essential that response actors are aware of the importance of trust and enact appropriate actions to ensure that trust is maintained and an integrated component throughout the emergency response.

## Data Availability

Due to the qualitative nature of the data, the confidentiality agreements signed and the ease with which respondents might be identified based on the content of the transcripts, we are unable to make the interview transcripts publicly available. A copy of the topic guide and consent form can be requested from the corresponding author.

## Abbreviations

ACDC: African centre for disease control
CHW: Community health worker
NGO: Non-governmental organization
PPE: Personal protective equipment
PHSM: Public health and social measures
WHO: World health organization
